# Race/Ethnicity-Resolved Time Trends in United States ASD Prevalence Estimates from IDEA and ADDM

**DOI:** 10.1007/s10803-019-04188-6

**Published:** 2019-08-21

**Authors:** Cynthia Nevison, Walter Zahorodny

**Affiliations:** 1 Institute for Alpine and Arctic Research, University of Colorado, Campus Box 450, Boulder, CO 80309-0450 USA; 2 Rutgers University, New Jersey Medical School, Newark, NJ USA

**Keywords:** Autism Spectrum Disorder, Prevalence, Time trends, ADDM, IDEA, Race/ethnicity, Black, White, Hispanic

## Abstract

Race-specific time trends in Autism Spectrum Disorder prevalence are tracked among 3–5 year-olds and 8 year-olds identified by the U.S. Individuals with Disabilities Education Act (IDEA) and the Autism and Developmental Disabilities Monitoring (ADDM) Network, respectively. White ASD prevalence historically has been higher than other racial groups but plateaued for IDEA birth cohorts from ~ 2004 to 2007 before resuming its increase. Black and Hispanic IDEA prevalence increased continuously and caught up to whites by birth year ~ 2008 and ~ 2013, respectively, with black prevalence subsequently exceeding white prevalence in the majority of states. Plateaus in white prevalence occurred in some ADDM states for birth years 2002–2006, but IDEA trends suggest prevalence will increase across all racial groups in ADDM’s birth year 2008 report.

**Electronic supplementary material:**

The online version of this article (10.1007/s10803-019-04188-6) contains supplementary material, which is available to authorized users.

## Introduction

Autism spectrum disorders (ASD) are a complex set of disorders characterized by impairments in social interaction, communication and restricted or stereotyped behaviors (APA 2013). Historically, diagnosed ASD prevalence has been higher among white children than among black or Hispanic peers. Also, black and Hispanic children have been more likely than white children to have severe forms of autism and/or co-occurring intellectual disability (Jarquin et al. 2011; CDC 2018). One explanation for these findings is that autism has been underdiagnosed in some traditionally underserved children, especially those who have milder symptoms (Liptak et al. 2008).

The most recent report of the Autism and Developmental Disabilities Monitoring (ADDM) Network found that overall ASD prevalence was 1.68% among 8 year-olds born in 2006 (CDC 2018). This represented a 15% increase from the ASD prevalence of 1.46% in cohorts born in 2002 and 2004, which, in turn, was more than double the baseline ADDM Network prevalence estimate of 0.67% among cohorts born in 1992 (CDC 2007a, 2014, 2016). The recent ADDM report noted that the prevalence of ASD among black and Hispanic children was approaching the rate identified in white peers (CDC 2018). It was suggested that the narrowing gap between white children and peers of other races might account for some or much of the increase between the birth year 2006 and previous ADDM Network reports.

California Department of Developmental Services (CDDS) data extend further back in time than ADDM to birth years well before 1992 (CDDS 2003). A recent analysis of CDDS data indicated a dramatic increase in U.S. autism prevalence over the last 8 decades to present, by as much as 1000-fold from birth year 1931 to 2012 and 25-fold from 1970 to 2012 (Nevison et al. 2018). The U.S. Department of Education provides additional data on the ASD classification of children, under the auspices of the Individuals with Disabilities Education Act (IDEA) (Gurney et al. 2003; Shattuck 2006). State and local education authorities, acting on behalf of the IDEA, have tracked children ages 3–21 receiving special education services, beginning in 1991, going back to birth cohorts of the early 1970s. The availability of cohort-specific autism counts in both the CDDS and IDEA datasets over multiple, successive, years allows for “constant-age tracking” of ASD prevalence, among specific age groups. The ADDM network also uses a constant-age tracking method, over successive biannual reports, focusing specifically on 8 year-olds (CDC 2007a, b, 2009a, b, 2012, 2014, 2016, 2018), though recent work also has tracked prevalence among 4 year-olds (CDC 2019).

In this paper, we use constant-age tracking to describe race-specific trends among black, Hispanic and white children, and examine how race and ethnicity contribute to the total ASD trend across all races. We characterize the time trend in race-specific United States autism prevalence using the two best available datasets, which, in the IDEA dataset, extend most recently to 3 to 5 year-old children, born in 2012–2014. We pose the hypothesis that increased diagnosis of black and Hispanic children is responsible for the continued upward trend in ASD prevalence in recent years and consider what the IDEA 3–5 year-old data might portend for future ADDM reports and for ASD prevalence in the United States.

## Methods

### Autism Prevalence Data

Race-specific and overall trends in ASD prevalence in the United States are examined using the constant-age tracking approach, in which the prevalence of autism is tracked among 3 to 5 year-olds across 18 years of annual IDEA reports and among 8 year-olds across eight biannual ADDM Network reports. The IDEA and ADDM reports are described in greater detail below and the complete datasets used in our study are provided in Supplementary Files S2-4. Since the relevant information was without identifying information and since the datasets were aggregated by age at the state level, this project did not require institutional review and approval.

### Individuals with Disabilities Education Act (IDEA) Autism Classification Data

The Individuals with Disabilities Education Act (IDEA) requires the collection of special education enrollment counts for 13 specific child disability categories, including autism. IDEA is federally mandated and conducted under the authority of the U.S. Department of Education, but it allows individual states discretion in determining student eligibility under the special education categories. Determination of whether a student qualifies for services under an autism classification is made by district-level professionals, using state-level guidelines in concert with the student’s parents and teachers (MacFarlane and Kanaya 2009).

Autism classification counts were obtained from the IDEA Part B database for each of the 50 United States (http://www.ideadata.org and later https://www2.ed.gov/programs/osepidea/618-data/state-level-data-files/part-b-data/child-count-and-educational-environments/). From 2000 to 2007, annual autism counts by state for children ages 3–5 years were partitioned into five race/ethnicity groups: American Indian or Alaska Native (AIAN), Asian or Pacific Islander, Black (not Hispanic), Hispanic, and White (not Hispanic). In 2008–2009, the annual reports shifted to categorizing seven race/ethnicity groups (with many states still using the old five race/ethnicity groups), in which group 2 above was split into (1) Asian and (2) Native Hawaiian or Other Pacific Islander, and a seventh group, Two or More Races, was added. From 2010 and 2017 all states had transitioned to the seven race/ethnicity groups, with a major change in the formatting of the Part B IDEA report starting in 2012. In this study, we focus on autism classification of 3 to 5 year-olds using the following race/ethnicity categories: (1) Black (not Hispanic), (2) Hispanic, and (3) White (not Hispanic), and (4) All races, where category 4 encompasses all race/ethnicity groups. We include the combined Asian/Native Hawaiian or Other Pacific Islander category in our Supplemental Spreadsheets but do not present it in the main text both because it is small in most states and also to avoid confusion over the splitting of the category into two separate groups around 2008. We neglect the AIAN group because it is very small and the Two or More Races group because it was not consistently available in the IDEA annual reports across the full 2000–2017 time span of this study.

Autism prevalence was computed by dividing the IDEA counts by total statewide race-specific public school kindergarten populations (i.e., 5 year-olds), multiplied by 3, as provided by the National Center for Education Statistics (NCES) (http://nces.ed.gov/ccd/elsi/). The factor of 3 in the prevalence calculation assumes that the 3 and 4 year-old populations (which are not comprehensively available from NCES) are similar to the kindergarten population. The NCES data are available in annual reports for all races as a whole and partitioned into five race/ethnicity groups for the 2000–2007 reports and seven race/ethnicity groups for 2008–2016 reports, consistent with the partitioning of the IDEA data described above. We also assumed that the 2016 race-specific kindergarten populations were a good approximation of the 2017 kindergarten populations (which were not yet available from NCES at the time of this study).

The full datasets of race-specific IDEA autism classification counts and NCES kindergarten populations are provided in Supplementary Files S2 and S3, respectively. Both datasets are presented for all 50 United States plus Washington, D.C. We also computed overall race-specific autism prevalence estimates for the U.S. from 2000 to 2017 by summing all race-specific autism counts from all available states for each year with non-blank data and dividing by the sum of the race-specific NCES public school populations in those states. Race-specific autism classification data were available from at least 48 states for each year from 2000 to 2017.

### Autism and Developmental Disabilities Monitoring (ADDM) Network

The Autism and Developmental Disabilities Monitoring (ADDM) Network is an ongoing, active, multiple source ASD surveillance system established by the Centers for Disease Control (CDC) in 2000 and conducted in multiple select U.S. regions to provide estimates of ASD prevalence among 8 year-old children. Reports of ASD prevalence are available biannually for birth years from 1992 to 2006, for a total of eight reports (CDC 2007a, b, 2009a, b, 2012, 2014, 2016, 2018; Zahorodny et al. 2014). ADDM ASD cases are determined by systematic review and analysis of information contained in existing professional evaluations conducted for developmental health and special education purposes. In some states, ADDM researchers have access only to health records and not education records. (Note: Separate health-only and health-plus-education surveys were reported by Maryland in 2004 and Colorado in 2000. We used only the health-plus-education data in this study.) ADDM uses U.S. Census-based data for the age cohort denominators needed to compute prevalence. ADDM Network estimates through birth year 2006 were based on the DSM-IV criteria and encompass all ASD subtypes, including Autistic Disorder (AD), Pervasive Developmental Disorder-Not Otherwise Specified (PDD-NOS) and Asperger’s Disorder (APA 1994). Over the lifetime of the Network, ADDM included parts of 18 different states. However, the states surveyed are not consistent from report to report and the number of counties referenced in each report is also somewhat variable.

In addition to tracking site specific and overall ASD prevalence, ADDM presents race/ethnicity-specific prevalence estimates for each participating state in three categories: white (non-Hispanic) black (non-Hispanic) and Hispanic across all reports (in Table 3 for the 2012–2014 reports and Table 2 for all other reports). Asian/Pacific Islander ASD prevalence is also presented in all but the 2000 report, but this category is blank for a number of states/years. The total survey population in each race/ethnicity category is also provided in Table 1 of the ADDM reports. Using the information described above, race-specific prevalence estimates and least squares linear regression slopes were calculated for each group, over time. We split these linear regressions into two periods, encompassing the first set of five reports (birth years 1992–2000) and the three most recent reports (birth years 2002–2006), due to strong non-linearity across these time periods among some race/ethnicity groups in a number of states. This presentation focuses on 9 out 18 total ADDM states that met two criteria: (1) Participation in the most recent report (i.e., survey year 2014, birth year 2006) and (2) participation in at least five reports over the lifetime of the ADDM Network.

We also calculated proportional contributions for each race using total 8 year-old populations (rather than race-specific) denominators to calculate prevalence. For these calculations, we backed out the total number of ASD counts for each race group by multiplying the reported race-specific prevalence rate by the total race-specific survey population, then divided those ASD counts by the total 8 year-old population. Finally, we note that ADDM data are resolved for each state by gender or by race (see Tables 2 and 3 in the biannual reports), but not by both simultaneously. IDEA 3–5 year old data are only resolved by race, not by gender. Thus, it was not possible to examine race trends by gender with the data used in this study.

## Results

### Autism Trends Among 3 to 5 Year-Olds from IDEA

**Fig. 1:**
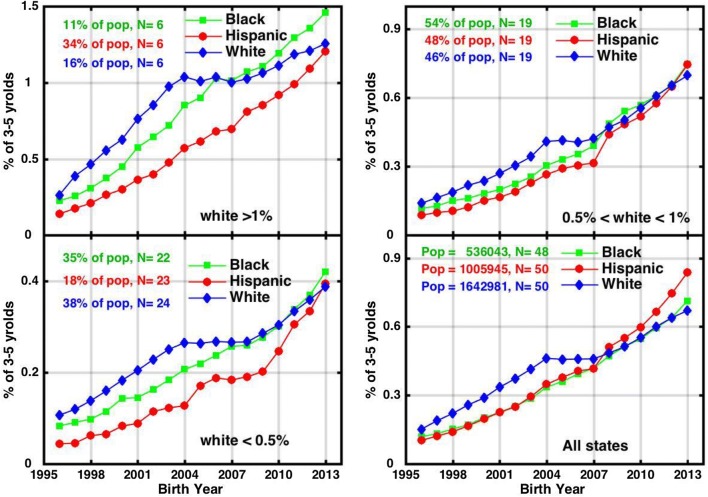
Race-specific overall IDEA Autism classification prevalence trends tracked among black, Hispanic and white 3 to 5 year-old children, sorted into 3 categories according to the absolute value of white prevalence for children born in 2013: high prevalence > 1% (upper left), medium prevalence < 0.5% < 1% (upper right), low prevalence > 0.5% (lower left), and all states nationwide (lower right). The number (N) of states in each category is shown in the panel. The total kindergarten population of each race/ethnicity group is listed in the lower right panel. The high, medium and low prevalence panels list the percentage of the total race/ethnicity population that falls into each category. Mean birth year on the x-axis assumes a mean age of 4 in the aggregate 3–5 year-old age group

The race-specific trend curves in the IDEA dataset for 3 to 5 year-olds are shown for all individual states in Supplementary Figure S1. Different states vary substantially in the overall magnitude of autism classification in this young age group, ranging from 1 to 2% in states like California, Massachusetts and Maine and < 0.4% in other states like Arizona, Missouri and Oklahoma. The overall nationwide trend among white children shows that ASD prevalence climbed steeply for the 1996–2004 birth cohorts and then plateaued around 0.46% nationwide between birth years 2004–2007. After the 2007 cohort, white ASD prevalence resumed its climb, reaching 0.67% nationwide by 2013 (Fig. 1).

The mid 2000s plateau in white prevalence is evident in many individual states, although the IDEA data are also erratic in a number of states (Figure S1). When the data are sorted according to absolute value of white prevalence in the most recent report year 2017 (i.e., < 0.5%, 0.5–1%, > 1%), the mean of each of these subgroups looks similar to the nationwide mean in showing a flattening of the white prevalence trend between about birth year 2004–2007 (Fig. 1).

Hispanic and black ASD prevalence estimates were substantially lower than white prevalence across the early IDEA reports in most states. However, in more recent years, ASD prevalence among black children caught up, by about birth year ~ 2008, and thereafter exceeded prevalence among white children in the majority (~ 30) of states (Supplementary Figure S1). Meanwhile ASD prevalence among Hispanic children caught up to prevalence among white children by birth year 2013 on average (Fig. 1a–c) and even exceeded white prevalence in 18–20 states in the last three IDEA reports (Supplementary Figure S1). In the nationwide mean (Fig. 1d), ASD prevalence among Hispanic children began growing the fastest of the three groups starting around birth year 2007, reaching 0.84% by birth year 2013, compared to about 0.7% among white and black children. However (as discussed further below) a disproportionate share of the Hispanic kindergarten population (34%) lives in the six states, which include California, that report high prevalence of > 1% in the 3–5 year-old age group. In contrast, only 11% of the black population and 16% of the white population live in those states.

**Fig. 2:**
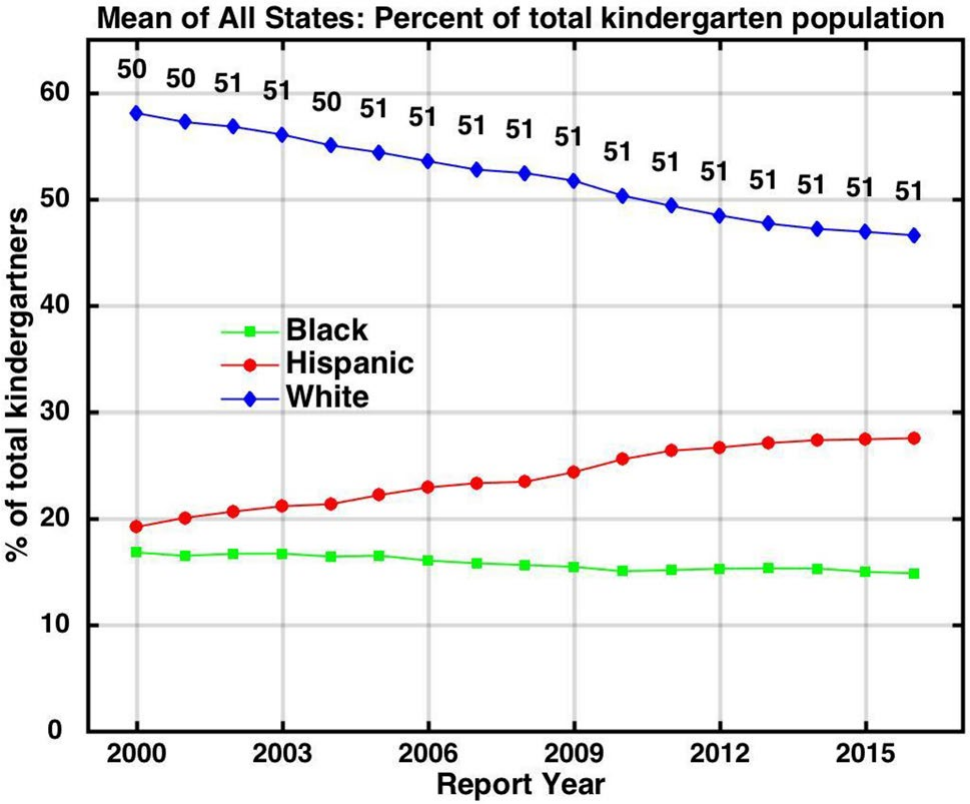
Trends in the percentage contributions of black, Hispanic and white children to the U.S. kindergarten population. The numbers tagged to each data point show the number of U.S. states plus the District of Columbia that had non-blank data for each year of the prevalence calculation

Unlike white prevalence, Hispanic and black prevalence, as reflected in IDEA data, did not show an obvious plateau in the mid 2000s but, rather, increased continuously (Fig. 1). Meanwhile, the Hispanic portion grew from 19% of the total U.S. kindergarten population reported by NCES in 2000 to 27.5% in 2016 (Fig. 2). Concurrently, the white portion declined from 58% to less than 47%. Blacks also declined slightly from about 17 to 15% of all kindergartners. Asian, Native American and mixed race children accounted for the balance of the population.

**Fig. 3:**
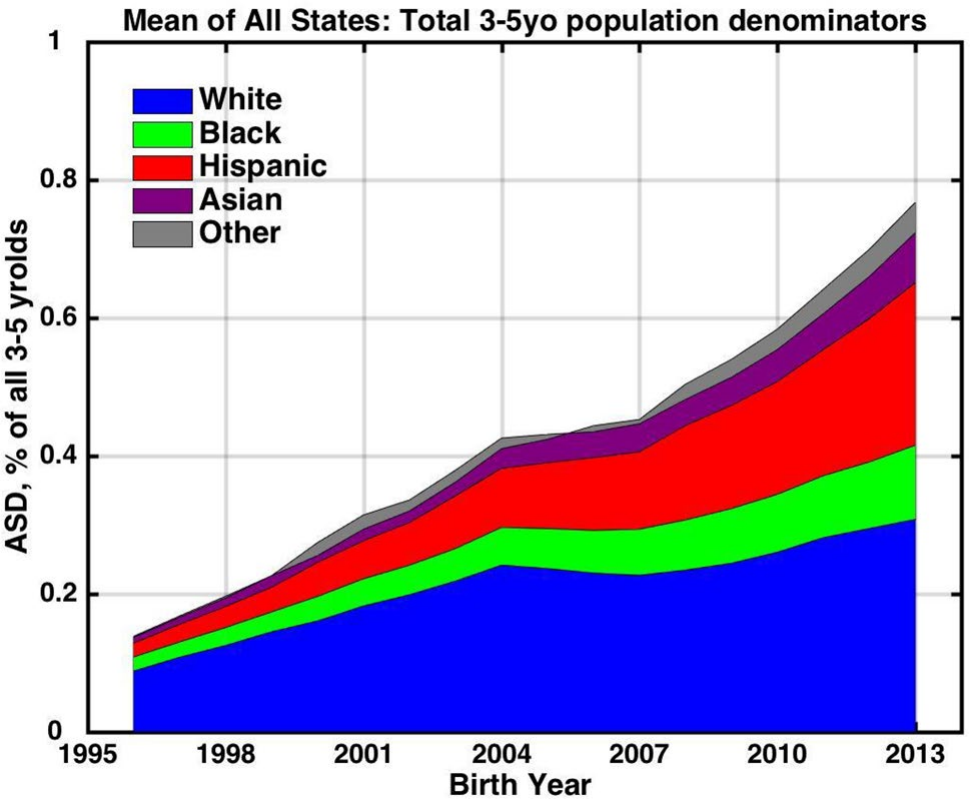
IDEA Autism prevalence trends tracked among different race/ethnicity groups for the 3 to 5 year-old age group, showing the contribution of each group to overall prevalence

The net result of the changing racial demography and race-specific ASD prevalence trends (Figs. 1, 2) was a continuous increase in overall 3–5 year-old prevalence from birth year 1996 to 2013, albeit with some flattening during the time of the white plateau (Fig. 3). Overall ASD prevalence increased by 0.63% (from 0.14 to 0.77%) from birth year 2000 to 2017 among 3 to 5 year-olds. White and Hispanic children each accounted for just over 1/3 of that increase, black children for about 1/6 of the increase and all other racial groups for the remaining fraction.

The states divide relatively evenly in terms of which race group shows the fastest specific rate of increase over the full set of 2000–2017 IDEA reports. Using least squares linear regression to quantify the trend slopes, the fastest growth occurred among white, Hispanic and black children in 11, 15 and 24 states, respectively (Figure S1). However, when the linear fit was restricted to the reports from 2012 to 2017, the race-specific growth rate in autism prevalence was fastest among Hispanic children in the (clear) majority (n = 30) of states. Only eight states showed fastest race-specific growth among white children.

In the earlier 2000–2011 IDEA reports in our study, total ASD counts are available in age-resolved annual reports for 3, 4 and 5 year-olds. For 2012–2017, only aggregated age groups (e.g., 3–5, 6–11, 12–17) are available. In all years, only 3–5 year-olds and 6–21 year-olds are resolved by race. The mean age of the total 3–5 year-old ASD population in the fully age-resolved 2000–2011 reports was 4.3, which we rounded off to ~ 4 in Figs. 1, 2, 3. Only two states (New Jersey and West Virginia) did not report any 3 or 4 year-olds, such that their 3–5 year-old autism counts consisted entirely of 5 year-olds. We assumed that ~ 4 continued to be the mean age in the 2012–2017 reports.

### ADDM 8 Year-Old ASD Prevalence in Nine States

**Fig. 4:**
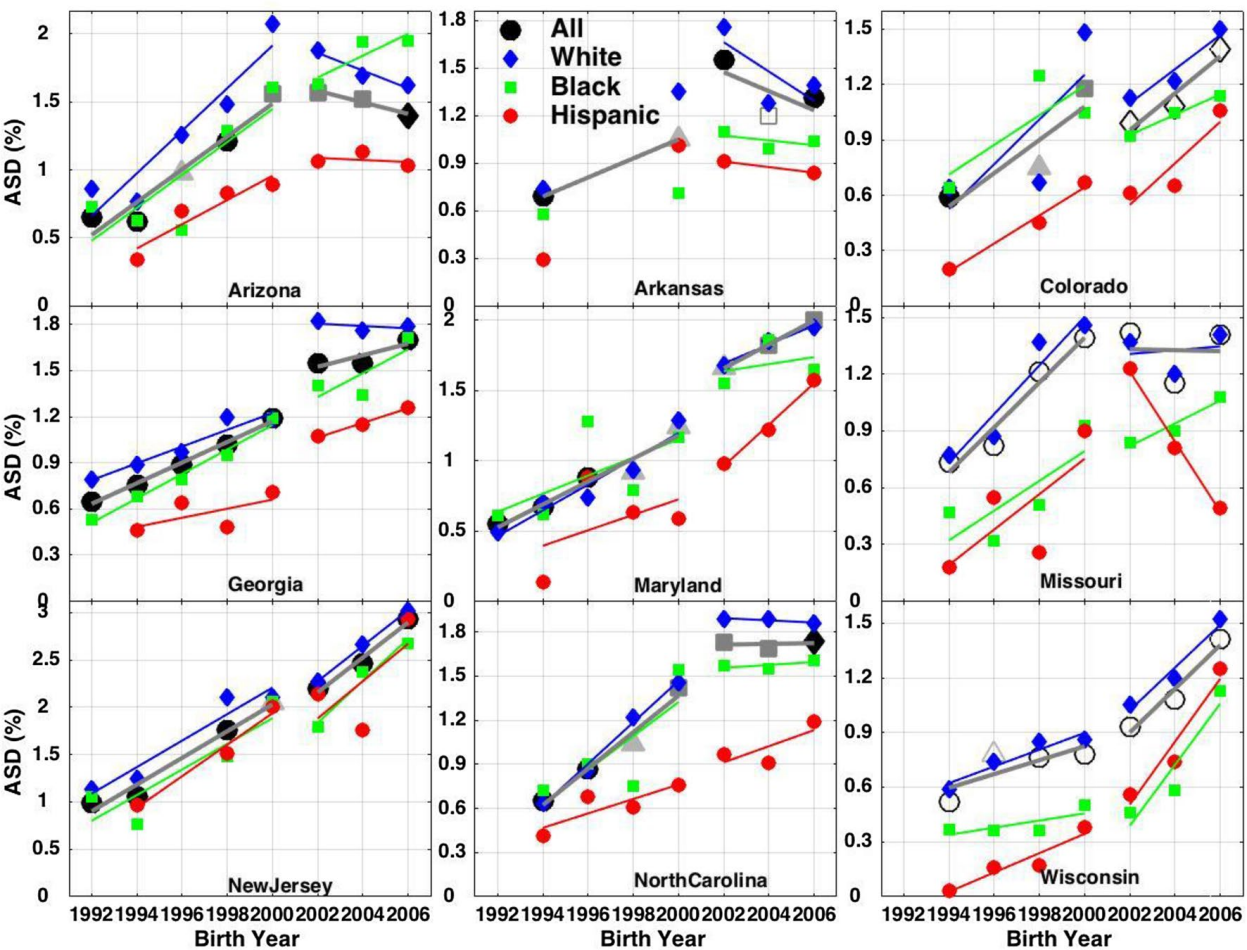
ASD prevalence, using race-specific denominators for black, Hispanic and white children, tracked among 8 year-olds, over 8 biannual ADDM Network reports. Overall prevalence among 8 year-olds is also tracked using up to 4 different black or grey symbols to denote shifts and inconsistencies in the number of counties sampled within each state in successive reports. In addition, the overall ADDM data are plotted as solid symbols for ASD prevalence derived from analysis of both health and education records and as open symbols when only health records were available for the majority of the site’s survey population. Least squares linear regression slopes for each race/ethnicity group are shown, with separate regressions performed for birth years 1992–2000 and 2002–2006

In the nine states with the longest participation in the ADDM Network, ASD prevalence among 8 year-olds varied in both absolute value of prevalence and in race/ethnicity trends (Fig. 4). White ASD prevalence was always higher than Hispanic prevalence across all eight cycles of monitoring and almost always higher than black prevalence, except for a handful of years in a few states. Over the first five ADDM reports (birth years 1992–2000), ASD prevalence increased in all states and for all races. In contrast, across the most recent three ADDM reports (birth years 2002–2006), white prevalence increased in four states (Colorado, Maryland, New Jersey and Wisconsin), remained flat in three (Georgia, Missouri and North Carolina) and decreased in two states (Arizona and Arkansas). During this same period, Hispanic prevalence estimates increased in six out nine states and remained flat or decreased in Arizona, Arkansas and Missouri. Meanwhile, black ASD prevalence increased in six states, but remained relatively flat or decreased in Arkansas, Maryland and North Carolina.

Georgia and New Jersey were the two ADDM states with the most consistent and complete ASD case ascertainment, i.e., with access to both health and education records and a relatively consistent survey population. Both states had highest prevalence among whites, but different race-specific trends. In New Jersey, the rate of increase over birth years 2002–2006 was continuous and similar across white, black and Hispanic children. In contrast, in Georgia, white prevalence plateaued over birth years 2002–2006 while Hispanic and black prevalence continued to increase, with black prevalence nearly catching up to white prevalence by 2006, but Hispanic prevalence still lagging.

The white percentage of the ADDM survey population has fluctuated and/or declined substantially in most states (Supplementary Figure S2). In Georgia, for example, white children declined from over 42% of the survey population in birth year 1992 to only 30% by birth year 2006 and in North Carolina they declined from 58.5 to 50%. Simultaneously and in contrast, the Hispanic percentage of the ADDM survey population has increased substantially in almost all states. For example, Hispanic children increased from 10 to 19% of the 8 year-old population of Georgia and from 8 to 18% in North Carolina. The percentage of black children in the ADDM population has fluctuated or declined in some states, but generally remained more constant than that of white or Hispanic children.

Over the history of the Network, white children accounted for the large majority of all ASD cases (Supplementary Figure S3). However, the combination of the plateau in race-specific white prevalence and the declining fraction of the white population reduced the white contribution to increasing ASD prevalence in recent years. Conversely, the combination of increasing race-specific Hispanic ASD prevalence and the increasing Hispanic population fraction enhanced their contribution to increasing ASD prevalence.

## Discussion

One of the most prominent features of the IDEA 3–5 year-old dataset is the plateau in white ASD prevalence over birth years in the mid 2000s followed by a renewed increase in prevalence after birth year 2007. This plateau might suggest a stabilization of the environmental drivers of ASD in the mid-2000s followed by a new or increasing environmental insult after 2007 (Nevison 2014). Hispanic and black prevalence both increased more or less continuously across birth years 1996–2013, without an obvious plateau, although these groups also had lower prevalence than whites throughout most of this time. It is therefore possible that the mid-2000s plateau is not evident for these races because they were still catching up from historical underascertainment during those years, due to more effective outreach to black and Hispanic communities. However, the finding that black prevalence has exceeded white prevalence in the majority of states since birth year 2009, and that Hispanic prevalence also recently has surpassed white prevalence in a third of all states, suggests that additional factors beyond catch-up and access to services may be involved.

Changes in reporting also may have influenced the observed trends. A major shift in the formatting of the IDEA reports occurred in 2012 (birth year ~ 2008), around the same time that the plateau in white prevalence ended and resumed its rise. To our knowledge, though, the determination of autism classification did not change in 2012 when the IDEA reports were reformatted. Furthermore, the uptick in prevalence around birth year 2007–2008 is also seen in the California DDS dataset (Nevison et al. 2018), which did not undergo a change in reporting protocol at that time. Notably, prevalence did not plateau during birth years in the mid 2000s in the CDDS dataset, but it did grow at a slower rate than during the birth years of the early 2000s. The CDDS trends are not inconsistent with the IDEA data, given that whites comprised only one quarter of California’s kindergarten population over those years, such that a plateau in the white-specific trend may have been overshadowed by ongoing increases among other racial groups.

A recent race-resolved analysis of CDDS cases, age 7 years or older, provides important insight into this issue (Pearl et al. 2019). That analysis indicates that the annual rate in growth of autism prevalence among privately-insured whites indeed slowed down, by more than a factor of 5, to a near plateau between birth years 2000–2010. Hispanic ASD prevalence (both privately or publicly insured) also experienced a near plateau, albeit more briefly, between birth years 2003–2006. However, ASD prevalence among publicly-insured whites (considered “lower income” by the study) increased strongly over birth years 2000–2010, ending at 1.2% in birth year 2010, compared to 0.9% among privately-insured whites. Black ASD prevalence (both privately or publicly insured) also increased strongly over this time frame, ending nearly 40% higher than overall white prevalence by birth year 2010.

The publication of DSM-5 in November, 2013 (APA 2013) may have affected the IDEA trends, although the influence of the new diagnostic guidelines on IDEA is unclear given the discretion afforded to states to determine an autism classification for special education purposes. DSM-5 formally defined the term Autism Spectrum Disorder (ASD), encompassing but no longer distinguishing between milder and more severe subtypes. Assuming DSM-5 was widely adopted by the time of the 2014 IDEA report (birth years 2009–2011), the resumed growth in ASD prevalence, which occurred around birth year 2007, would have predated this diagnostic change. Nevison et al. (2018) reached a similar conclusion about CDDS trends, i.e., that the renewed uptick in prevalence predated the change to DSM-5.

An additional possibility is that the continuous growth in ASD prevalence among 3–5 year-old black and Hispanic children throughout the 2000–2017 span of IDEA reports reflects a shift toward earlier age of diagnosis among those groups, which can create an artefact upward trend in constant-age tracking data (Hertz-Picciotto 2009). We were not able to assess this possibility directly from the available IDEA data, which do not partition 3, 4 and 5 year-olds separately by race/ethnicity. However, recent ADDM Network findings do not support a specific shift toward earlier diagnosis among blacks and Hispanics. The most recent ADDM report found that the median age of earliest known ASD diagnosis remained consistent over the history of the ADDM Network at 52 months and did not differ significantly by race/ethnicity (CDC 2018).

It is notable that access to health care among blacks and Hispanics improved over the decade that black and Hispanic prevalence caught up to and/or surpassed white prevalence. For children in the 1990s on Medicaid, blacks and Hispanics were shown to receive their first autism diagnosis 1.5–2.5 years later on average than the mean white diagnosis age of 6.3 years (Mandell et al. 2002). Similarly, a 2003/2004 survey found that being black, Latino, or poor was associated with decreased access to services (Liptak et al. 2008). In contrast, a 2013/2014 survey found little inequality between non-Hispanic whites and other races (Augustyn et al. 2019). Much of this improvement may be attributed to the State Children’s Health Insurance Program (CHIP), which has increased access to timely preventive care, specialty care and prescription medications (Liptak et al. 2008). CHIP began in 1998 and was expanded in 2009. CHIP has preferentially boosted insurance coverage for Hispanic and African American populations in particular, leading to increased health services for those populations (Kaiser Family Foundation 2019).

Hispanic autism prevalence has only caught up with white prevalence very recently in the IDEA 3–5 year-old dataset, achieving similar values around birth year 2012 or 2013 in the low, medium and high prevalence states (Fig. 1a–c). In the nationwide mean, Fig. 1d suggests that Hispanic prevalence may now be growing even faster than white or black prevalence, although this may be an artefact of the fact that a disproportionate share of the Hispanic kindergarten population (34%) lives in the six states that report high prevalence of > 1% in the 3–5 year-old age group. Within that high prevalence group itself (Fig. 1a), Hispanic prevalence was substantially lower than black or white prevalence over almost the entire history of the IDEA reports, catching up to whites only in birth year ~ 2013 on average. However, when the states are examined individually, Hispanic prevalence exceeded white prevalence in over a third of states by birth year 2012–2013 (Supplementary Figure S1). Furthermore, a recent study of individuals ages 7 or older found that Hispanic autism prevalence in California surpassed white prevalence in birth year 2010, at which time the Hispanic prevalence rate was 1.14% compared to 1.07% for whites (Pearl et al. 2019).

Black IDEA autism prevalence estimates caught up somewhat earlier to white prevalence, around birth year 2008 (Fig. 1). Subsequently, it has increased at a similar or higher rate than white prevalence, particularly in the high prevalence category states (Fig. 1a), and has exceeded white prevalence in the majority of states since the 2013 IDEA report (birth year ~ 2009). Thus, the IDEA data suggest that prevalence among black children already exceeds white prevalence in the majority of states and that prevalence among Hispanic children may be trending in that direction too. However, the IDEA data also suggest that these recent race/ethnicity trends are likely not yet apparent in available ADDM data, which thus far have extended only through birth year 2006.

The IDEA age 3 to 5 year-old autism dataset, which encompasses all states, and the population-based ADDM 8 year-old ASD dataset are the two primary ongoing sources of race-resolved ASD prevalence information available in the U.S. (IDEA also tracks race-resolved autism counts among 6 to 21 year-olds, but we considered this too broad an age range to provide meaningful constant-age tracking trends.) The two datasets cover overlapping birth year intervals (1996 ± 1 to 2013 ± 1 for IDEA and 1992–2006 for ADDM). IDEA data extend up to 8 years farther into recent time both because they sample a population that is ~ 4 years younger and because there is a 4 year lag in the publication of ADDM data, e.g., the 2014 survey of 8 year-olds born in 2006 was published in 2018 (CDC 2018). Due to their timely annual availability, IDEA autism classification data may provide a bellwether of forthcoming ASD prevalence trends in the ADDM Network. We advance this idea with the caveat that the IDEA autism counts for 3–5 year-olds significantly underrepresent the ADDM ASD estimates for 8 year-olds (CDC 2019).

In addition, some important differences and discrepancies between the IDEA and ADDM datasets should be acknowledged. The core purposes and definitions of the datasets are different, as are the methodologies and the level of detail characterizing the cases. ADDM is population-based while IDEA may be considered universal because it encompasses the vast majority of school age children. The ages of subjects vary, as do the roles of parents and education or health care professionals in instigating the evaluation for ASD. Recent studies generally have not found significant differences among whites and non-whites in health care-based screening rates and referral practices for toddlers (Rea et al. 2019) or in wait time to care, referral and final diagnosis following evaluation by pediatricians for school-age children (Augustyn et al. 2019). Recent studies also generally have found only limited differences between whites and non-whites in the receipt of school-based physical and occupational therapy (Bilaver et al. 2019). However, these researchers all noted the difficulty of separating effects due to socioeconomic status from those due to race/ethnicity and called for further research into lingering race or wealth-based disparities in access to health and education resources.

Over the duration of the ADDM Network, each biannual report has brought shifts in which and how many states were included. In addition, more recently, at some sites with a history of consistent participation, the number of surveyed counties changed across reports, as did access to records (i.e., health-plus-education versus health-only records). These inconsistencies complicate the Network’s ability to track trends in ASD prevalence. As the ADDM investigators commented most recently, “Comparisons with earlier ADDM Network surveillance results should be interpreted cautiously because of changing composition of sites and geographic coverage over time.” (CDC 2018; see also Supplementary File S4).

The inconsistencies of sampling complicate the interpretation not only of the overall ASD trend but also of the race/ethnicity-specific trends. Across the nine most stable ADDM states, white-specific ASD prevalence declined over the most recent three reports in both Arizona and Arkansas, but these were states with inconsistent sampling in which black and/or Hispanic-specific prevalence estimates also decreased. Three other ADDM Network states showed plateaus in white prevalence, but among these, Missouri also showed a sharp decrease in Hispanic prevalence and North Carolina reported a plateau in black ASD prevalence. Only in Georgia did white ASD prevalence stabilize over birth year 2002–2006, while Hispanic and black prevalence contemporaneously continued to increase. However, Hispanic prevalence in birth year 2006 was still 30% lower than white prevalence in Georgia (1.26% compared to 1.79%) (CDC 2018). Indeed, as of birth year 2006, white and Hispanic children still had the highest and lowest, respectively, race-specific rates of ASD in almost all ADDM states, including Georgia, and IDEA data suggest the gap may not close until birth year 2013.

The most recent ADDM Network report (CDC 2018) suggested that white ASD prevalence had largely stabilized and that the 15% increase in prevalence, from 1.46% in birth year 2002–2004 to 1.68% in birth year 2006, was driven by the catch-up of Hispanics and blacks who had been historically underascertained. This interpretation appears substantially based on the ASD prevalence findings from Georgia, which is the only ADDM state whose findings fully support the hypothesis. Notably this hypothesis is not supported by the findings from New Jersey, which, after Georgia, is the ADDM state with the most complete and geographically consistent ascertainment. In New Jersey, white ASD prevalence continued to increase over the birth year period 2002–2006, at a rate comparable to the increases in Hispanic and black prevalence.

The declining white population and increasing Hispanic population may also influence trends in ASD prevalence, both in past and future ADDM reports. Using total population (rather than race-specific) denominators, the white-absolute ASD prevalence declined in five out of nine states (Supplementary Figure S3). In two of these, Arizona and Arkansas, white-specific ASD prevalence also decreased, as discussed above. However, the white-absolute declines in the remaining three states (Georgia, North Carolina and Maryland) resulted from the decreasing white fraction of the school population combined with flat or only slowly increasing white-specific prevalence.

The plateau in white autism prevalence in 3 to 5 year-old in IDEA data from birth years 2004-2007 occurred during roughly the same period as the flattening of white prevalence seen in 8 year-olds in some ADDM sites, although differences in age and likely completeness of ascertainment, as well as inconsistencies in the ADDM protocol, complicate the comparison. The renewed growth in autism prevalence among white children, beginning around birth year 2007 and the ongoing growth in black and Hispanic prevalence observed in the IDEA dataset suggest that similar increases in ASD prevalence across all race and ethnicity groups, including whites, may be observed in the next ADDM report, which will focus on 8 year-olds born in 2008. Indeed, a recent report on 4 year-olds by the Early ADDM Network found an increase of 40% in ASD prevalence among children born in 2014 compared to children born in 2010 (from 2.0 to 2.8%) in New Jersey (CDC 2019). While the other two participating sites, Arizona and Missouri, reported no clear trend among 4 year-olds, these sites also show ambiguous trends in the 8 year-old counts (Fig. 4), possibly due to lack of access to education records (Missouri) and/or frequent changes and inconsistencies in the ascertainment region (Arizona).

## Conclusion

Historically, autism prevalence has been reported higher among white children than among other race/ethnicity groups. Recently, however, Hispanic and black prevalence in the IDEA 3–5 year-old autism dataset has “caught up” to and, in the case of blacks, surpassed white prevalence in the majority of states. The fastest growth in prevalence reflected in the IDEA dataset has been observed among Hispanic children, with Hispanic autism prevalence exceeding white prevalence in one-third of states in the 2013 birth cohort. Meanwhile, white ASD prevalence in the IDEA dataset appeared to plateau for birth cohorts in the mid 2000s in many states, but resumed its upward climb among children born around or after 2007. A plateau in white prevalence was also observed among 8 year-olds for birth years 2002–2006 in some ADDM states, but was inconsistent across the 9 states with the longest record of ADDM participation. As of birth year 2006, white children still had the highest race-specific rate of ASD in almost all ADDM states, while Hispanic children had the lowest race-specific rate. The overall trend in ASD prevalence reflects the combined influence of differences in race-specific prevalence convolved with the declining white and increasing Hispanic fractions of the U.S. school population. The available IDEA 3–5 year-old autism count, which extends to the 2013 birth cohort, suggests that the forthcoming ADDM report (birth year 2008/study year 2016) will show ASD increases across all race/ethnicity groups.

## Electronic supplementary material

Below is the link to the electronic supplementary material.

Supplementary material 1 (PDF 1413 kb)

Supplementary material 2 (XLSX 37 kb)

Supplementary material 3 (XLSX 40 kb)

Supplementary material 4 (XLSX 27 kb)

## References

[CR1] American Psychiatric Association (1994). Diagnostic and statistical manual of mental disorders.

[CR2] American Psychiatric Association (2013). Diagnostic and statistical manual of mental disorders.

[CR3] Augustyn M, Silver EJ, Blum N, High P, Roizen N, Stein REK (2019). DBP evaluations in DBPNet sites: Is race/ethnicity a significant factor in care?. Journal of Developmental and Behavioral Pediatrics.

[CR4] Bilaver LA, Havlicek J (2019). Racial and ethnic disparities in autism-related health and educational services. Journal of Developmental and Behavioral Pediatrics.

[CR5] California Department of Developmental Services. (2003). Autistic spectrum disorders. Changes in California Caseload. An update: 1999 through 2002. Sacramento, CA

[CR6] Centers for Disease Control and Prevention (2007). Prevalence of Autism Spectrum Disorders—Autism and developmental disabilities monitoring network, Six sites, United States, 2000. Morbidity and Mortality Weekly Report.

[CR7] Centers for Disease Control and Prevention (2007). Prevalence of Autism Spectrum Disorders—Autism and developmental disabilities monitoring network, 14 sites, United States, 2002. Morbidity and Mortal Weekly Report.

[CR8] Centers for Disease Control and Prevention (2009). Brief update: Prevalence of Autism Spectrum Disorders (ASDs)—Autism and developmental disabilities monitoring (ADDM) network, United States, 2004. Morbidity and Mortality Weekly Report.

[CR9] Centers for Disease Control and Prevention (2009). Prevalence of Autism Spectrum Disorders—Autism and developmental disabilities monitoring network, United States, 2006. Morbidity and Mortality Weekly Report.

[CR10] Centers for Disease Control and Prevention (2012). Prevalence of Autism Spectrum Disorders—Autism and developmental disabilities monitoring network, 14 Sites, United States, 2008. Morbidity and Mortality Weekly Report.

[CR11] Centers for Disease Control and Prevention (2014). Prevalence of autism spectrum disorder among children aged 8 years—Autism and developmental disabilities monitoring network, 11 sites, United States, 2010. Morbidity and Mortality Weekly Report.

[CR12] Centers for Disease Control and Prevention (2016). Prevalence and characteristics of Autism Spectrum Disorder among children aged 8 years—Autism and developmental disabilities monitoring network, 11 Sites, United States, 2012. MMWR Surveillance Summaries.

[CR13] Centers for Disease Control and Prevention (2018). Prevalence and characteristics of Autism Spectrum Disorder among children aged 8 years—Autism and developmental disabilities monitoring network, 11 Sites, United States, 2014. MMWR Surveillance Summaries.

[CR14] Centers for Disease Control and Prevention (2019). Prevalence and characteristics of Autism Spectrum Disorder among children aged 4 years—Early autism and developmental disabilities monitoring network, 7 sites, United States, 2010, 2012, and 2014. MMWR Surveillance Summaries.

[CR15] Gurney JG, Fritz MS, Ness KK, Sievers P, Newschaffer CJ, Shapiro EG (2003). Analysis of prevalence trends of autism spectrum disorder in Minnesota. Archives of Pediatrics and Adolescent Medicine.

[CR16] Hertz-Picciotto I, Delwiche L (2009). The rise in autism and the role of age at diagnosis. Epidemiology.

[CR17] Jarquin VG, Wiggins LD, Schieve LA, Van Naarden-Braun K (2011). Racial disparities in community identification of Autism Spectrum Disorders over time; Metropolitan Atlanta, Georgia, 2000–2006. Journal of Developmental and Behavioral Pediatrics.

[CR18] Kaiser Family Foundation, 2019, Total number of children ever enrolled in CHIP annually, https://www.kff.org/other/state-indicator/annual-chip-enrollment, accessed 31 Jul 2019.

[CR19] Liptak GS, Benzoni LB, Mruzek DW, Nolan KW, Thingvoll MA, Wade CM, Fryer GE (2008). Disparities in diagnosis and access to health services for children with autism: Data from the National Survey of Children’s Health. Journal of Developmental and Behavioral Pediatrics.

[CR20] MacFarlane JR, Kanaya T (2009). What does it mean to be autistic? Inter-state variation in special education criteria for autism services. Journal of Child and Family Studies.

[CR21] Mandell DS, Listerud J, Levy S (2002). Race differences in the age at diagnosis among Medicaid-eligible children with autism. Journal of the American Academy of Child and Adolescent Psychiatry.

[CR22] Nevison CD (2014). A comparison of temporal trends in United States autism prevalence to trends in suspected environmental factors. Environmental Health.

[CR23] Nevison C, Blaxill M, Zahorodny W (2018). California autism prevalence trends from 1931–2014 and comparison to national ASD data from IDEA and ADDM. Journal of Autism and Developmental Disorders.

[CR24] Pearl M, Matias S, Poon V, Windham G (2019) Trends in birth prevalence of autism spectrum disorder (ASD) in California from 1990 to 2010, by race-ethnicity and income, International Society for Autism Research (INSAR), 2019 annual meeting, Poster 31748, Montreal, Canada, May 4, 2019

[CR25] Rea KEM, Armstrong-Brine L, Ramirez L, Stancin T (2019). Ethnic disparities in Autism Spectrum Disorder screening and referral: Implications for pediatric practice. Journal of Developmental and Behavioral Pediatrics.

[CR26] Shattuck PT (2006). The contribution of diagnostic substitution to the growing administrative prevalence of autism in US special education. Pediatrics.

[CR27] Zahorodny W, Shenouda J, Howell S, Rosato NS, Peng B, Mehta U (2014). Increasing autism prevalence in metropolitan New Jersey. Autism.

