# Supplementary material for: Race/Ethnicity-Resolved Time Trends in United States ASD Prevalence Estimates from IDEA and ADDM
Source: J Autism Dev Disord. 2019 Aug 21;49(12):4721–30. doi: 10.1007/s10803-019-04188-6 (PMC6841650; doi:10.1007/s10803-019-04188-6)
Supplement: Supplementary file 1 — Supplementary material 1 (PDF 1413 kb) [file 10803_2019_4188_MOESM1_ESM.pdf]

Race/ethnicity-resolved time trends in United States ASD prevalence estimates from IDEA and ADDM

Supplementary File1: Figures S1-S3

Supplementary Figure S1. Race-specific time trends in Autism Spectrum Disorder (ASD) for blacks (green), Hispanics (red) and whites (blue) are plotted for all available states + D.C. using constant-age tracking of 3 to 5 year-olds over IDEA report years 2000-2017. This time span corresponds to birth years ~1996-2013, assuming a mean group age of 4. The data are plotted with slightly offset colors between report years 2000-2011 and 2012-2017 to denote a major shift in the formatting of the IDEA Part B reports starting in 2012. Due to the wide range among states in reported prevalence in this young age group, which may reflect differences in resource availability and diagnostic practices, the states are sorted based on the absolute value of prevalence among whites in 2017:

1. High group: States in which white prevalence > 1% (6).

2. Medium group: States in which 0.5% < white prevalence < 1% (19).

3. Low group: States in which white prevalence < 0.5% (24).

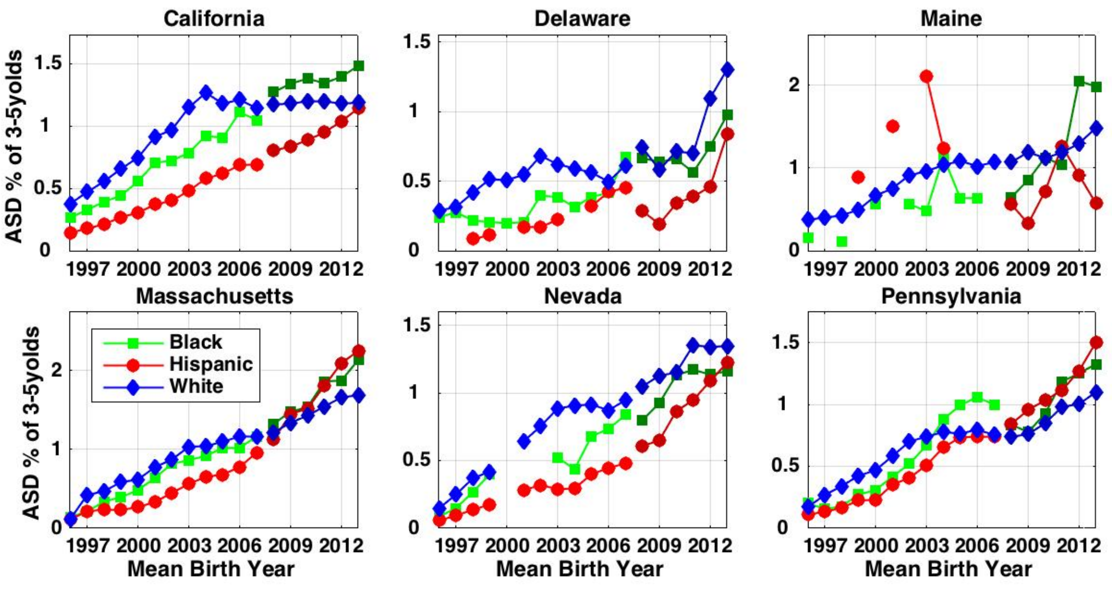

High group: States in which white prevalence > 1%

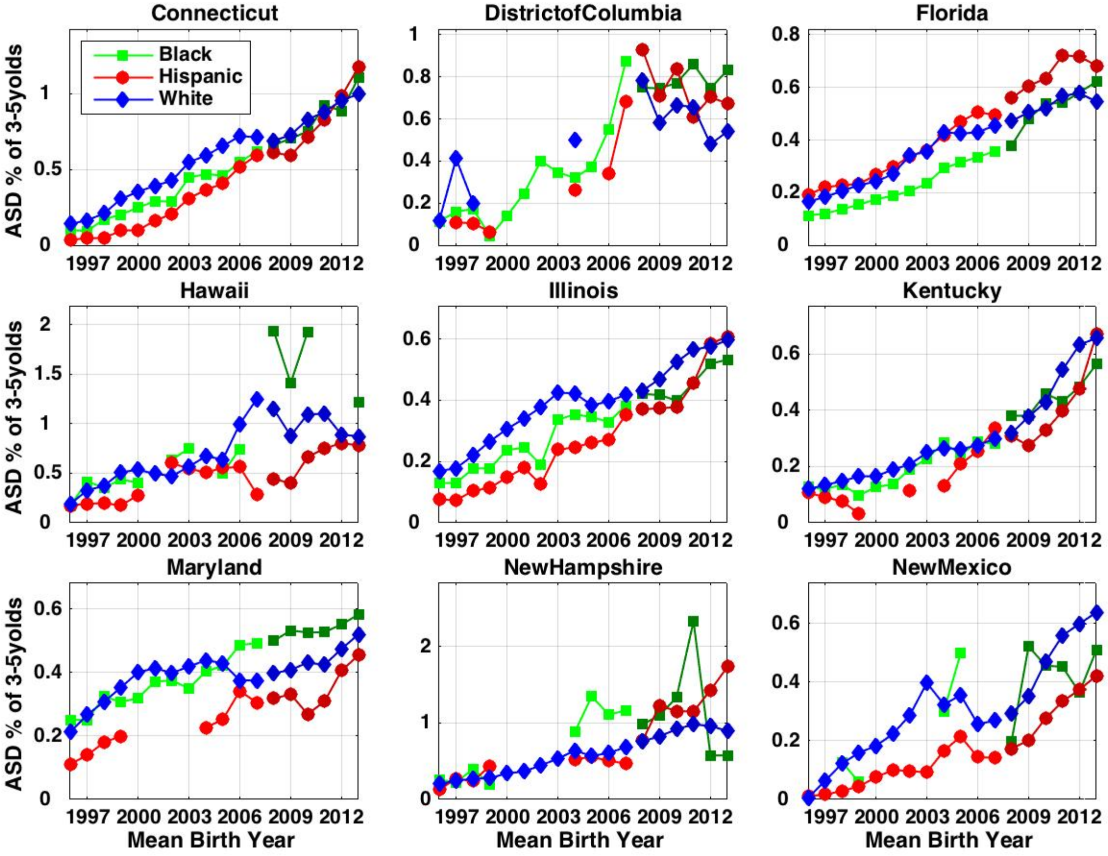

Medium group: States in which 0.5% < white prevalence < 1% (page 1).

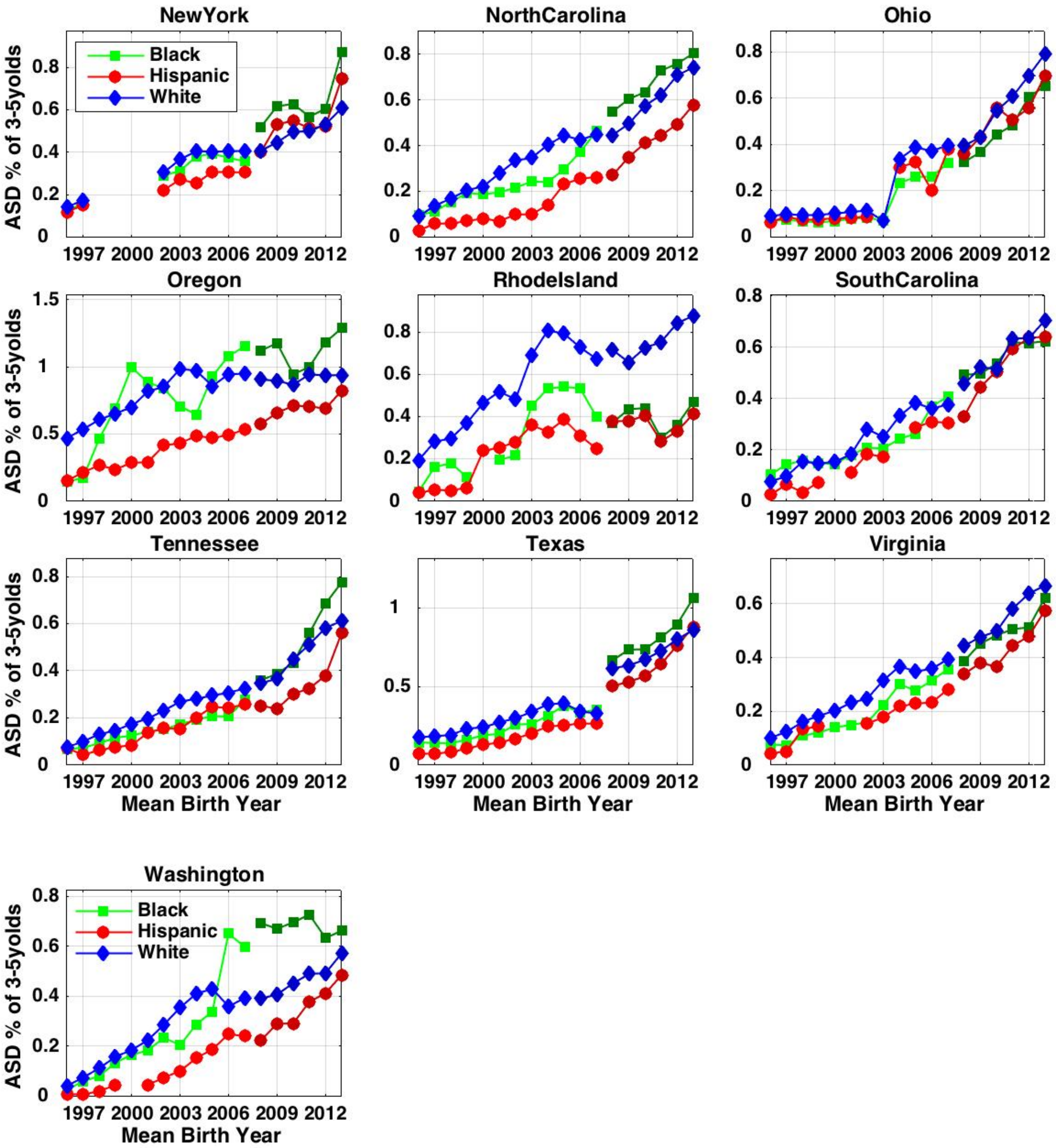

Medium group: States in which 0.5% < white prevalence < 1% (page 2).

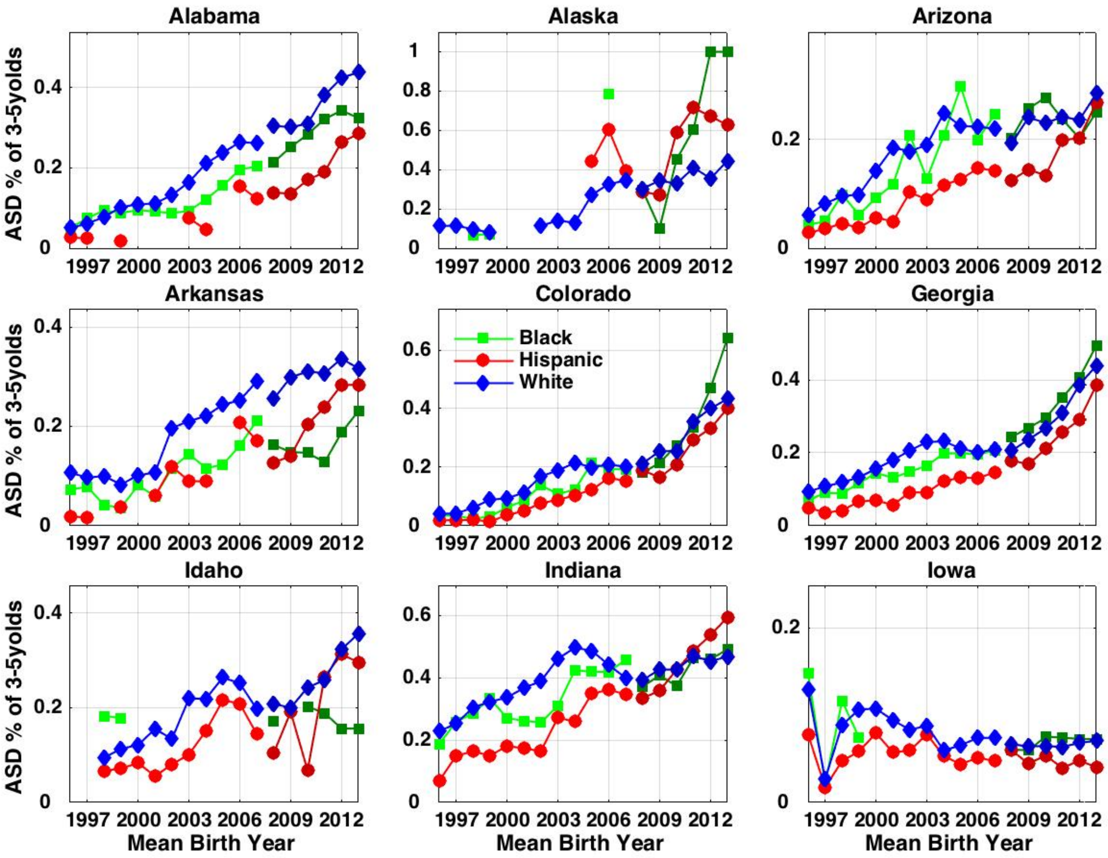

Low group: States in which white prevalence < 0.5% (page 1).

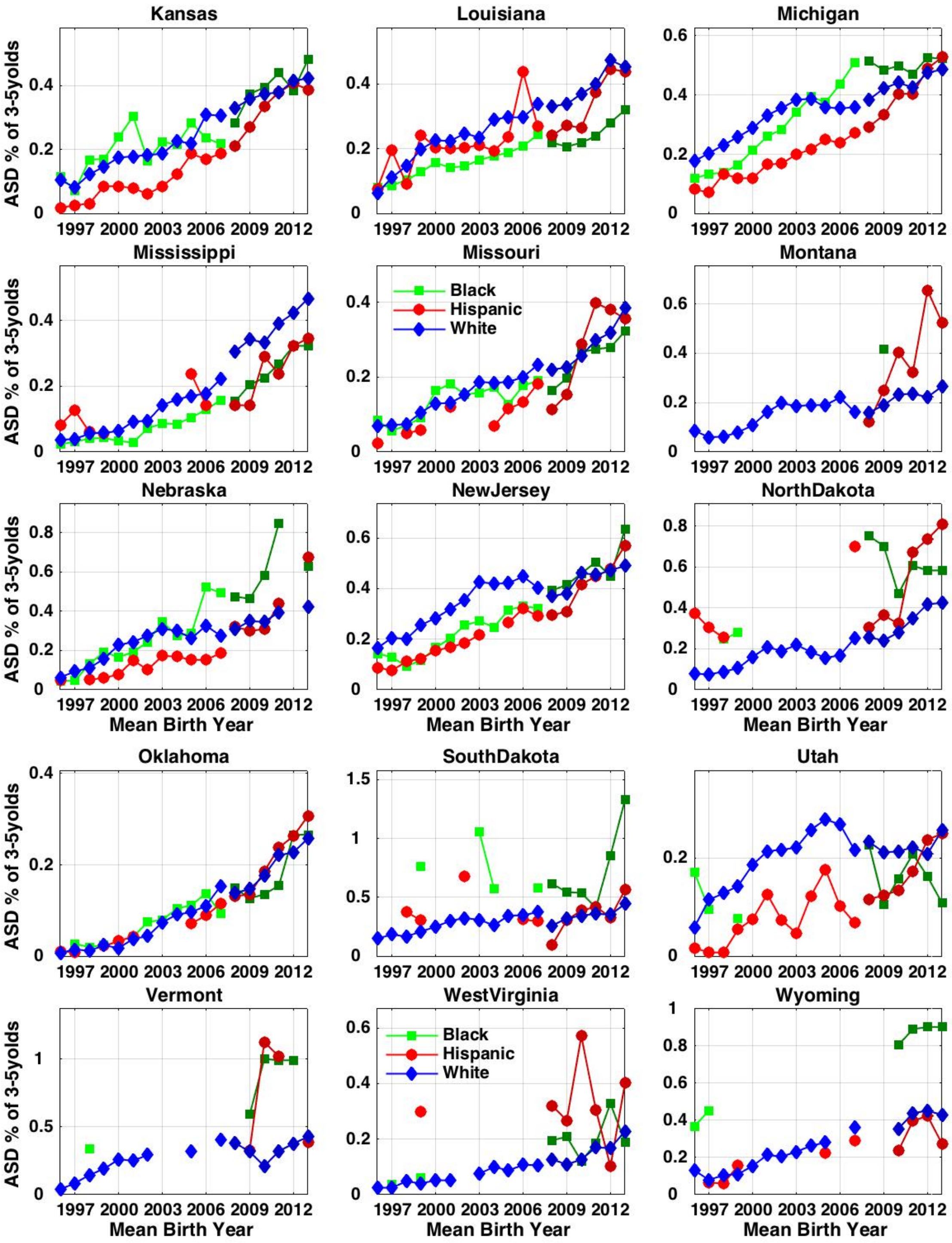

Low group: States in which white prevalence < 0.5% (page 2).

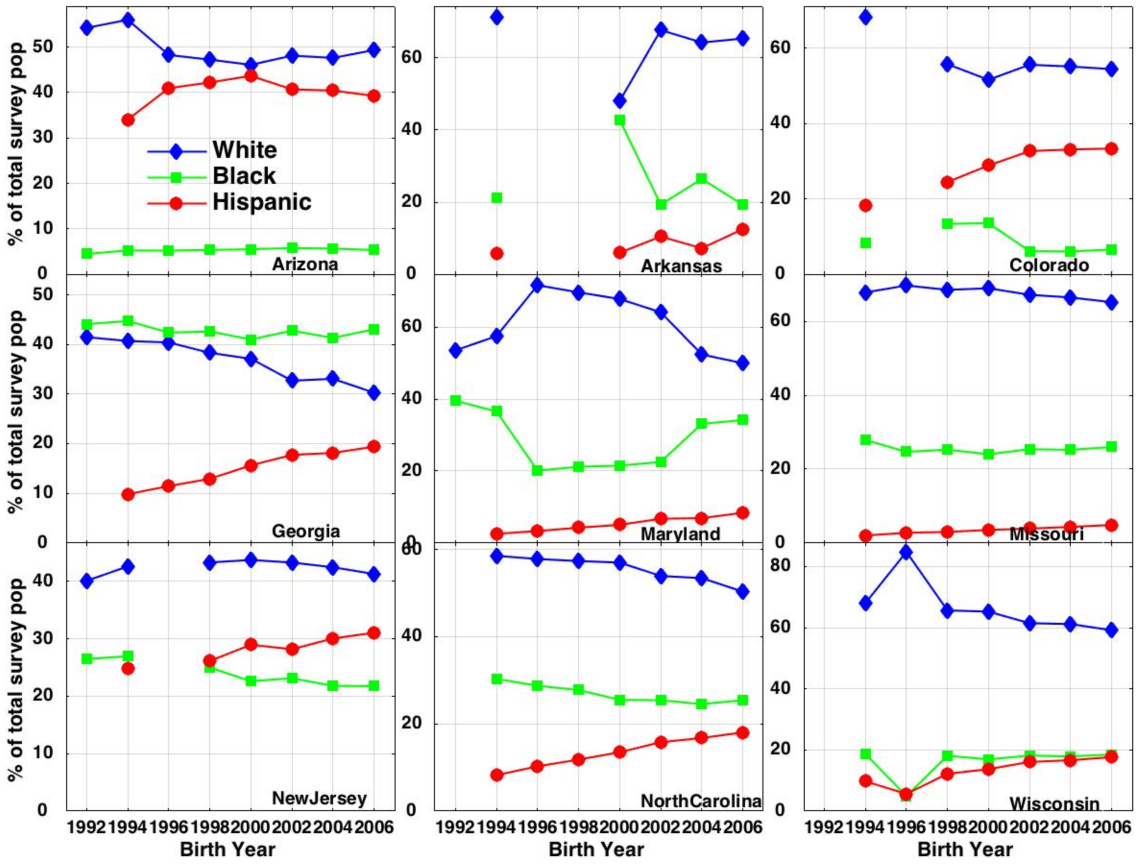

Supplementary Figure S2. Trends in the percentage contributions of black, Hispanic and white children to the total survey population in each ADDM state.

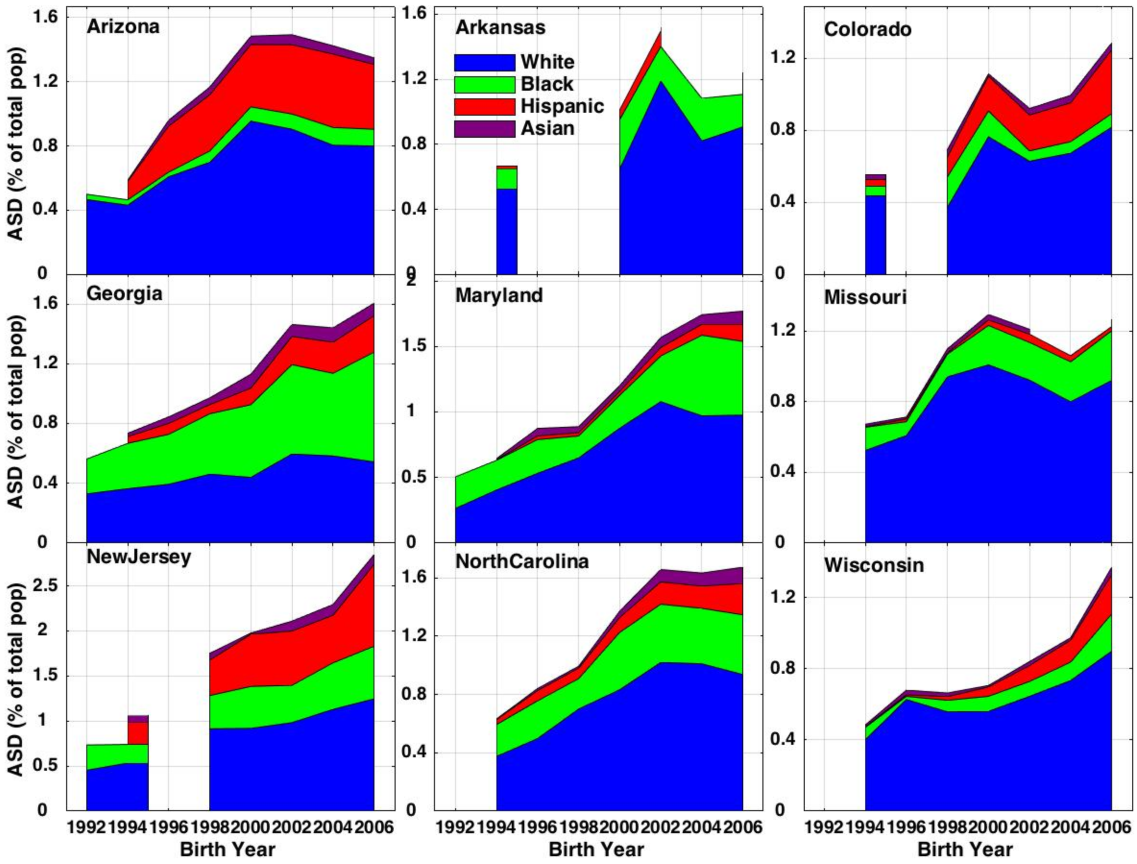

Supplementary Figure S3. ASD prevalence, using total 8 year-old survey population denominators in each state, tracked among 8 year-olds, over 8 biannual ADDM Network reports spanning birth years 1992-2006, showing the contribution of each race/ethnicity group to overall prevalence.
